# Apatinib: A Novel Antiangiogenic Drug in Monotherapy or Combination Immunotherapy for Digestive System Malignancies

**DOI:** 10.3389/fimmu.2022.937307

**Published:** 2022-06-29

**Authors:** Haosheng Li, Haiyan Huang, Tao Zhang, Haoran Feng, Shaodong Wang, Yaqi Zhang, Xiaopin Ji, Xi Cheng, Ren Zhao

**Affiliations:** ^1^ Department of General Surgery, Ruijin Hospital, Shanghai Jiao Tong University School of Medicine, Shanghai, China; ^2^ Shanghai Institute of Digestive surgery, Ruijin Hospital, Shanghai Jiao Tong University School of Medicine, Shanghai, China

**Keywords:** Apatinib, angiogenesis, digestive system malignancies, tumor microenvironment, immunotherapy

## Abstract

Digestive system malignancies are one of the primary causes of cancer-related death. Meanwhile, angiogenesis has been proved to play an important role in the process of cancer neovascularization. Apatinib, a novel targeted antiangiogenic molecule, could generate highly selective competition in the vascular endothelial growth factor receptor-2, involved in tumor progression and metastasis. It has been implied as a promising cancer treatment agent that can prevent tumor cell proliferation meanwhile inhibit tumor angiogenesis. Furthermore, completed clinical trials demonstrated that apatinib could prolong the progression-free survival and overall survival in advanced gastric cancer and primary liver cancer. Recent studies revealed that apatinib had a synergistic effect with immunotherapy as a second-line and third-line treatment regimen for some other cancers. In this review, we summarize the pharmacological properties of apatinib and the latest clinical application in chemotherapy-refractory patients with advanced digestive system cancer. Based on the comparable survival results, the molecular mechanisms of apatinib are prospective to include the antiangiogenic, apoptosis-inducing, and autophagy-inducing properties in the corresponding signaling pathway. Treatment of apatinib monotherapy or combination immunotherapy remains the optimal option for patients with digestive system malignancies in the future.

## Introduction

Cancer is still one of the principal causes of death, among which digestive system malignancies (DSMs) account for the majority of morbidity and mortality. According to the latest statistics from the American Cancer Society, there will be approximately 338,090 Americans diagnosed and 169,280 will die from DSMs in 2021 (1). Malignant tumors of the digestive system, such as gastrointestinal cancer, liver cancer, cholangiocarcinoma, and so on, account for a large proportion of DSMs. Gastric cancer ranked the third in the world of cancer-related causes of death (2). The fourth leading cause of cancer death in the world is primary liver cancer, and cholangiocarcinoma is also one of the highly malignant tumors (3). Moreover, colorectal cancer (CRC) is one of the most common cancers worldwide and accounts for nearly 8.5% of total cancer mortality (4). In addition, esophageal cancer is the fourth leading cause of cancer-related deaths among malignant tumors worldwide (5). To an aging population, harmful dietary habits and lifestyles can increase the risk of DSMs onset in high-income countries. Advancements in a better understanding of the pathophysiology provide new treatment options for advanced and late-stage cancer diseases including individualized treatment (6). To date, receptor tyrosine kinases (RTKs) are transmembrane glycoproteins in the process of angiogenesis that connect with extracellular ligands and cellular growth factors. Many important physiological processes are activated and regulated, including cell proliferation, cell growth, cell migration, cell differentiation, and apoptosis. RTKs are also associated with pathological conditions including cancer, metabolic and immune diseases. The development of antiangiogenic agents targeting the vascular endothelial growth factor (VEGF) signaling pathway plays a crucial role in the treatment of cancer in recent years, including bevacizumab, ramucirumab, sorafenib, and apatinib (7).

Apatinib, a novel, small molecule, selective tyrosine kinase inhibitor (TKI), could inhibit multiple tumor-related kinases (TRKs), such as vascular endothelial growth factor receptor-2 (VEGFR-2), and induce apoptosis, suppressing tumor proliferation in multiple tumors (8). It was first verified to prolong the progression-free survival (PFS) and overall survival (OS) in advanced gastric cancer in a phase III clinical trial. Thus it was approved by the National Medical Products Administration (NMPA) of China in 2014 as an alternative anti-neoplastic drug available for patients with advanced gastric cancer or gastroesophageal junction (GEJ) carcinoma (9). Furthermore, Apatinib exhibits promising anti-tumor activities in various tumors, including hepatocellular carcinoma, breast cancer, and non-small-cell lung cancer (10–12). Additionally, Apatinib could sensitize resistant tumor cells to chemotherapy drugs meanwhile reverse the multiple drug resistance (MDR) caused by ATP-binding cassette transporters proteins (13).

Inhibition of angiogenesis is an important target of tumor therapy while angiogenesis is one of the immune evasion mechanisms (14, 15). Immunotherapy has become an integral part of cancer treatment, demonstrating unprecedented clinical benefits in a variety of malignancies, including patients with aggressive, metastatic, and drug-resistant malignancies (16, 17). However, mounting evidence shows that only a subgroup of patients benefit, and tumors may adopt additional mechanisms for immune destruction (16). Therefore, there is an increasing interest in enhancing antitumor immunity with novel combinatorial therapeutic strategies of immunotherapy (18, 19). Growing evidence suggests that persistent angiogenesis contributes to immune evasion by inducing a highly immunosuppressive tumor microenvironment (TME) (20). Due to persistent pro-angiogenic signaling pathways, morphologically abnormal tumor vasculature results in additional blood perfusion and oxygenation, leading to hypoxic areas and low pH in the TME (21). Angiogenesis also impedes leukocyte infiltration and inhibits dendritic cell maturation and increases intratumoral numbers of myeloid-derived suppressor cells and regulatory T cells in order to affect immune responses (22). Thus tumor angiogenesis promoting immune barrier for circulating leukocytes has been well established, and an increasing number of immune cells exhibit the dual ability to promote immunosuppression and angiogenesis (23, 24). This impelled researchers to design a dual inhibition study of antiangiogenic and anti-barrier compounds, which could potentially synergize for the treatment of DSMs (25). Therefore, the use of antiangiogenic agents to rebuild TME appears to be an effective way to improve immune efficacy (15, 26). The fact that the ‘immune-excluded’ tumor phenotype has described the presence of T cells in the tumor stroma but not in its parenchyma is also identified (27). Moreover, it has also been found that VEGF inhibits T cell development and function, as well as promotes T cell exhaustion by upregulating immune checkpoints (28). Indeed, the promise of an antiangiogenic drug in monotherapy or combination immunotherapy has been established.

All these shreds of evidence support apatinib to be a rather promising antiangiogenic agent for DSMs. Here in this review, we aim to provide an overview of the structure, pharmacodynamic, and pharmacokinetics of apatinib. Selected preclinical and clinical progress of monotherapy or combined use of antiangiogenic drugs and immunotherapy are also summarized. The inclusion criteria of trials are completed with NCT registration numbers and full data published in open-access journals. We highlight the possible molecular mechanisms meanwhile clinical relevance underlying the therapeutic potential of Apatinib in cancer treatment. Together, evidence contributes to a better understanding of angiogenesis therapy, thus achieving synergistic effects and reducing side effects.

## Structure, Pharmacodynamic, and Pharmacokinetics

Apatinib (trade name Aitan^®^), molecular formula of C_25_H_27_N_5_O_4_S, is an orally administered small-molecule receptor TKI with a molecular weight of 473.178 Da, first developed by Advenchen Laboratories, LLC (Northridge, CA, USA). The chemical formula of apatinib is N-[4-(1-cyano-cyclopentyl) phenyl]-2-(4-pyridylmethyl) amino-3-pyridine carboxamide derived from PTK787/ZK222584 (Valatinib) (29).

When stimulated by VEGF signaling, the carboxy-terminal tail and kinase-insert region of VEGFR‐2 are specifically auto-phosphorylated for the SH2 domains of various signaling molecules (29). Therefore, downstream signaling pathways such as ERK, PKC, and PLC are successively activated, leading to the peak pro-angiogenic effect. Apatinib could bind to the specific domain of the receptor and consequently inhibit subsequent effects of cell proliferation (30). Apatinib could also block c-Kit, platelet-derived growth factor receptor (PDGFR)-β, Ret, and c-Src in a concentration-dependent manner (29). Moreover, apatinib can suppress tumor growth *via* stimulating endoplasmic reticulum (ER) stress and protective autophagy, inducing apoptosis by multiple cellular signaling pathways (31, 32).

The recommended apatinib intake dose is 850 mg per day in China for the treatment of patients with advanced gastric cancer or GEJ carcinoma (9). Pharmacokinetic studies revealed that the mean time to peak plasma concentrations (C_max_) of apatinib was 3~4 hours, and the mean half-life (T_1/2_) was 9 hours following single 500, 750, or, 850 mg doses, meanwhile, the concentrations in plasma increased with dose (33). Interestingly, dose-normalized exposure could decrease with increasing doses in patients with advanced gastric cancer, suggesting non-linear dose proportionality. Based on a study on healthy volunteers and cancer patients, the latter exhibited delayed absorption and slower clearance as a result of renal or hepatic impairment (34). In the dosing groups of 850 mg (n=8), the C_max_ and AUC_24_ values showed high interindividual variability with a geometric mean of 2,833 ng/mL and 21,975 ng·h/mL. In addition, steady-state conditions of eleven patients were achieved by day 6, and no accumulation during 56 days of once-daily dosing with multidose cohort (33). The absorption and elimination processes in healthy volunteers and cancer patients with solid tumors were complexly exhibited by a two-compartment model with mixed first- and zero-order absorption together with first-order elimination. The analysis estimates of the absolute clearance (CL/F) of apatinib were 57.8 L/h, and its apparent volume in a stable condition was 112.5 L. The best feature of the nonlinear dose proportionality was its sigmoidal maximum effect (E_max_) function of relative bioavailability (34). The main pathways of apatinib biotransformation are E- and Z-cyclopentyl-3-hydroxylation, N-dealkylation, pyridyl-25-N-oxidation, 16-hydroxylation, deoxygenation, and O-glucuronidation after 3-hydroxylation. After being metabolized mainly by cytochrome P450 (CYP) 3A4/5 and, to a lesser extent, by CYP2D6, CYP2C9, and, CYP2E1, nine major metabolites were identified through comparison with the standard reference (35). Within 96 hours after administration, the total recovery rate of the administered dose was 76.8%, meanwhile, 69.8% and 7.02% of the administered dose were excreted through feces and urine, respectively (36). A negligible amount of unchanged apatinib was detected in the urine, indicating that the systemically available administered dose was extensively metabolized (33). The excellent properties of apatinib make it a promising drug for clinical translation with a sum of 408 relevant clinical trials underway.

## Clinical Efficacy in Advanced Gastric Cancer

### Monotherapy or Combination Chemotherapy of Apatinib

Based on preclinical studies and phase I data, the main outcome of the randomized, placebo-controlled, double-blind, phase II trial and the phase III trial (NCT00970138) was PFS, while OS was the other coprimary primary outcome in the phase III trial. Secondary endpoints included objective response rate (ORR), disease control rate (DCR), quality of life (QoL), and toxicity (9, 10). In the phase II trial, 144 patients in this study who had failed at least two chemotherapy regimens were divided into three groups, including placebo (group A, n = 48), lapatinib 850 mg once a day (group B, n = 47), or apatinib 425 mg twice a day (group C, n = 46). The results demonstrated that, compared with placebo, treatment with apatinib significantly prolonged the median PFS and OS. The median OS time for group A was 2.50 months (95% CI, 1.87 to 3.70 months) and the median PFS time was 1.40 months (95% CI, 1.20 to 1.83 months), while the median OS time for groups B and C were 4.83 months (95% CI, 4.03 to 5.97 months) and 4.27 months (95% CI, 3.83 to 4.77 months), and the median PFS time was 3.67 months (95% CI, 2.17 to 6.80 months) and 3.20 months (95% CI, 2.37 to 4.53 months), respectively. There was no statistically significant difference between the two different administration methods of apatinib. Concerning safety, patients in group B had fewer grade 3~4 adverse events, including hypertension, hand-foot syndrome, and diarrhea than patients in group C. Therefore, the phase II study recommended the dosing regimen of 850 mg once daily for the phase III trial (9). Among the 267 patients in the Phase III trial, the median OS of the apatinib group was significantly improved (6.5 months; 95% CI, 4.8 to 7.6 months) compared with the placebo group (4.7 months; 95% CI, 3.6 to 5.4 months) and the apatinib group significantly prolonged the median PFS(2.6 months; 95% CI, 2.0 to 2.9 months) in comparison to placebo (1.8 months; 95% CI, 1.4 to 1.9 months) (10).

At the time of the open-label, multicenter, noninterventional study of apatinib (NCT02668380), in a total of 337 patients, of which 62 (18.4%), 102 (30.3%), and 173 (51.3%) received first-line, second-line, and third-line or higher-line apatinib treatment respectively, were divided into three groups, low-dose Group (250 mg, n = 124), medium-dose group (425-500 mg, n = 198) and high-dose group (675~850 mg, n = 15). Findings of the study showed that there was no difference in the median OS (7.13 months; 95% CI, 6.17 to 7.93 months) and the median PFS (4.20 months; 95% CI, 4.60 to 4.77 months) among those three dose groups. Interestingly, the data also demonstrated that the treatment dose of apatinib received by patients with advanced gastric cancer was much lower than the dose used in clinical trials. The lower doses of apatinib (especially 425~500mg) produced the same clinical benefits compared with the higher dose drugs used in clinical trials with lower incidences of grade 3~4 adverse events (37). Coincidentally, in a prospective, multicenter observation study (NCT03333967), a total of 747 patients were eligible and received low-dose apatinib (500 mg or 250 mg per day) with the median PFS (5.56 months; 95% CI, 4.47 to 6.28 months) and the median OS (7.50 months; 95% CI, 6.74 to 8.88 months). Prospective studies suggested that a low-dose apatinib, which was lower than the previous phase III reported dose, was an effective regimen for advanced gastric cancer with tolerable or controllable toxicity (38).

The FAS analysis indicated that 54.9% of a total of 737 patients with advanced gastric cancer received the treatment of apatinib monotherapy, and 45.1% of patients were treated with combination therapy, apatinib plus chemotherapy, including 44.1% of patients in first-line treatment with apatinib, 28.2% in second-line treatment, and 27.7% in third-line and above treatment. In first-line treatment, the ORR of apatinib monotherapy and combination therapy groups was 9.09% and 16.42%, and the DCR was 78.41% and 89.29%, respectively. Compared with monotherapy, patients in combination therapy achieved significantly longer median PFS (6.18 months; 95% CI, 5.26 to 7.73 months versus 3.52months; 95% CI, 2.66 to 5.92 months) and median OS (8.72 months; 95% CI, 7.4 to 10.53 months versus 5.92months; 95% CI, 4.28 to 7.63 months). In second-line and third-line treatment, combination therapy showed a better trend in tumor response and survival outcomes in comparison to monotherapy. The safety of combination treatment, especially combined with paclitaxel, was tolerable, and no apatinib-specific adverse events had been reported (39). In a small retrospective study of 34 patients, the median PFS of the monotherapy group and concurrent apatinib and docetaxel therapy group was 2.5 months (95% CI, 1.99 to 3.01 months) and 4 months (95% CI, 3.29 to 4.71 months), with the median OS of 3.3 months (95% CI, 2.76 to 3.84 months) and 6 months (95% CI, 2.86 to 9.14 months), respectively. This study also found that grade 3~4 toxicities of neutropenia, thrombocytopenia, proteinuria, hyperbilirubinemia, hypertension, and fatigue were less in the combination therapy group than in the monotherapy group (40).

The combination of apatinib in neoadjuvant chemotherapy can turn unresectable advanced gastric cancer into resectable and obtain benefits in the long-term prognosis. Growing evidence suggested that preoperative chemotherapy could often convert initially unresectable gastric cancer into resectable cancers. In a retrospective study of 151 patients with unresectable gastric cancer receiving the combination of S-1 (a fluorouracil drug) and cisplatin or paclitaxel, the median OS time of 40 patients who received conversion surgery was significantly longer than 111 patients who underwent chemotherapy only (53 months versus 14 months) (41). For neoadjuvant chemotherapy, adding targeted drugs in the preoperative treatment had been recognized as an effective way to improve survival outcomes and response rates in patients with locally advanced gastric cancer. Therefore, a single-arm, open-label, phase II trial (NCT04208347) was conducted to evaluate the efficacy and safety of combining neoadjuvant chemotherapy with targeted drug for a total of 29 patients with advanced gastric adenocarcinoma. The patient received three preoperative cycles of neoadjuvant therapy (NAT) (3 weeks/cycle), including SOX (S-1: 80~120 mg/day on 2 weeks; oxaliplatin: 130 mg/m^2^ intravenously on day 1) and two consecutive cycles of orally administered apatinib (500 mg/day for 3 weeks) at one-cycle intervals, followed by surgery. After the resectability of tumors was assessed by CT, a standard D2 lymphadenectomy gastrectomy was performed and then the patients received three cycles of SOX adjuvant chemotherapy. Pathologic response rate (pRR), the primary end-point, was 89.7% (95% CI, 2.7 to 97.8%) with 28 patients who underwent surgery which was higher than the previously reported SOX regimen in Japan and the previous results in China due to the addition of apatinib in chemotherapy. All 29 patients could be evaluated for preoperative response evaluation with the ORR of 79.3% (95% CI, 60.3 to 92.0%), the DCR of 96.6% (95% CI, 82.2 to 99.9%), the margin-less resection rate of 96.6% (95% CI, 82.2% to 99.9%), and the pathological complete remission rate of 13.8% (95% CI, 1.2 to 26.3%). Concerning safety, the incidence of grade 3~4 adverse events occurred in 10 (34.5%) patients, and surgery-related complications were observed in 12 (42.9%) with the most common complication of fever with no treatment-related death occurring. All adverse events during the NAT period were tolerable and controllable, indicating that apatinib can be safely added to the SOX regimen before surgery (42). Another multicenter, prospective, single-group, open-label, nonrandomized controlled phase II trial (NCT03192735) provided a novel neoadjuvant chemotherapy option, apatinib combined with S-1 plus oxaliplatin, for patients with chemotherapy-refractory advanced gastric cancer. A total of 48 patients with M_0_ and clinical T_2_ to T_4_ or N^+^ diseases were eligible to receive apatinib (500 mg orally once a day for 3 weeks, and withdrawal in the last cycle) plus SOX, and then a standard D2 gastrectomy was performed within 2 to 4 weeks after the last cycle. The primary endpoint of the R0 resection rate in 40 patients who received surgery (95% had gastrectomy, and the rest had exploratory laparotomy) was 75.0% (95% CI, 60.4 to 86.4%). The radiological response rate was 75.0%, and 16 of 44 patients (36.4%) had T downstage. In the comparison of CT staging before and after treatment, 16 of 44 patients (36.4%) had T downstage, of which 10 patients (62.5%) dropped from T_4_ to T_3_, 4 patients (25.0%) from T_4_ to T_2_, 2 patients (12.5%) from T_4_ to T_1_, and 4 out of 41 patients (9.8%) had N downstage (N+ to N–). There were no grade 4 adverse events or preoperative death observed. Compared with patients who did not experience adverse events, patients with grade 3 adverse events had a significantly lower average number of preoperative cycles, indicating that the incidence of adverse events was not related to the cumulative exposure to the drug (43). Results from clinical trials demonstrated that neoadjuvant chemotherapy combined with apatinib achieved a better survival benefit in the unresectable advanced gastric cancer with acceptable safety.

### Combination Immunotherapy of Apatinib

In recent years, the use of anti-programmed cell death 1 (PD-1) in combination with TKI drugs to improve efficacy has attracted considerable attention (44). Although the additive or synergistic effect of this approach has been proved with strong scientific evidence, the acceptable level of toxicity is unknown (45). In 2019 Jianming Xu. et al. conducted a multicenter, single-arm, phase I dose-escalation and expansion study (NCT03463876) to evaluate the safety of the anti-PD-1 antibody camrelizumab (SHR-1210) in combination with apatinib as second-line, or later, therapy in patients with advanced HCC or GC/EGJC (46). A total of fifteen patients entered into the dose-escalation phase (Phase Ia), with patients (n=5 per cohort) receiving apatinib at doses of 125, 250, or 500 mg combined with SHR-1210 200 mg. 28 patients were enrolled in the dose-expansion phase (Phase Ib) and all 43 patients were conducted for safety assessment. In Phase Ia, no adverse events were reported in the 125 mg group. After 28 days of treatment, one patient in the 250 mg apatinib cohort developed grade 3 adverse events (elevated lipase without clinical symptoms of pancreatitis) and three patients in the 500 mg group had grade 3 immune-related pneumonitis. Among 23 evaluable GC/EGJC patients, median PFS was 2.9 months (95% CI, 2.5 to 4.2 months) and median OS was 11.4 months (95% CI, 8,6 to NR months), while the ORR was 17.4% (95% CI, 5.0 to 38.9%) and the DCR was 78.3% (95% CI, 56.3 to 92.5%). The convincing clinical efficacy obtained in this study was also higher than that of apatinib alone. However, apatinib with immunotherapy resulted in a slight increase in the incidence of some apatinib-related adverse events or serious adverse events, including hypertension and elevated alanine transaminase (ALT) and aspartate transaminase (AST) levels. According to Atkins and colleagues, camrelizumab may enhance these adverse events (47). Despite the small number of cases, the results may suggest that the combination treatment of camrelizumab and apatinib may present synergistic effects by stimulating the tumor-induced immunosuppressive microenvironment.

Camrelizumab (200 mg every 3 weeks, i.v.) in combination with capecitabine and oxaliplatin (CAPOX) followed by camrelizumab (200 mg once in 3 weeks, i.v.) plus apatinib (375 mg orally daily of every 3-week cycle) was evaluated in another multicenter, open-label phase II clinical trial (NCT03472365) as first-line combination therapy in patients with advanced gastric or gastroesophageal junction adenocarcinoma. The results of all 48 enrolled patients showed an ORR of 58.3% (95% CI, 43.2 to 72.4%), a median OS of 14.9 months (95% CI, 13.0 to 18.6 months), and a median PFS of 6.8 months (95% CI, 5.6 to 9.5 months). Grade ≥3 adverse events included treatment-related decreased platelet count (20.8%), decreased neutrophil count (18.8%), and hypertension (14.6%). One patient (2.1%) died due to abnormal liver function (48). Combinations of anti-PD-1 drugs and anti-angiogenesis agents are considered to induce T-cell activation and drive tumor cell responses to immune checkpoint blockade, resulting in a more adequate response to anti-PD-1 monotherapy (49). In an open-label, single-arm, phase II clinical trial (NCT04345783), a total of 24 patients with advanced gastric or gastroesophageal junction adenocarcinoma received 200 mg camrelizumab intravenously on day 1, 500 mg oral apatinib once daily, and specific dose of oral S-1 on days 1-14. Regardless of PD-L1 expression, the combination of camrelizumab, apatinib, and S-1 as a promising second-line therapy for cancer patients showed good antitumor activity and manageable toxicity (50).

The current selected clinical trial treatment efficacy data of apatinib are summarized in **Table 1**. In addition, a Phase III trial of apatinib in combination with immunotherapy (NCT03813784) is underway, and results will be reported in the near future.

**Table 1:** Selected clinical trials with apatinib in advanced gastric cancer.

Clinical trial identifier	Phase	Design	Type	Treatment	Primary endpoint	Results
NCT00970138	II/III	Randomized, placebo-controlled, parallel-arm	Interventional	Apatinib, 850 mg qd p.o. Apatinib, 425 mg bid p.o. Placebo bid p.o.	Median PFS (months); Median OS (months)	PhaseII: PFS: 3.67/3.20; OS: 4.83/4.27; PhaseIII: PFS: 2.6; OS: 6.5
NCT02668380	/	Prospective, open label, multicenter, noninterventional	Observational	Apatinib, 250 mg qd p.o. Apatinib, 425-500 mg qd p.o. Apatinib, 675-850 mg qd p.o.	Median PFS (months); Median OS (months)	PFS: 4.20; OS: 7.13 no difference among groups
NCT03333967	/	Prospective, multicenter, cohort	Observational	Apatinib, 250 mg qd p.o. Apatinib, 500 mg qd p.o.	Median PFS (months); Median OS (months)	PFS: 5.56; OS: 7.50
NCT04208347	II/III	Randomized, single-arm, open-label	Interventional	Apatinib, 500 mg qd p.o.; S-1, Oxaliplatin, every 2 weeks i.v.	pRR (%)	pRR: 89.7
NCT03192735	II	Multicenter, prospective, single-group, open-label, nonrandomized controlled	Interventional	Apatinib, 500 mg qd p.o.; SOX; Standard D2 gastrectomy	R0 resection rate (%)	R0 resection rate:75.0
NCT03463876	II	Single-arm, open-label, Multicenter,	Interventional	Apatinib, 125-500 mg qd p.o.; SHR-1210, 200mg every 2 weeks i.v.	Median PFS (months); Median OS (months); ORR (%); DCR (%)	PFS: 2.9; OS: 11.4; ORR: 17.4; DCR: 78.3
NCT03472365	II	Randomized	Interventional	Apatinib, 375mg qd p.o.; Camrelizumab, 200mg every 3 weeks i.v.; CAPOX	Median PFS (months); Median OS (months); ORR (%)	PFS: 6.8; OS: 14.9; ORR: 58.3
NCT04345783	II	Prospective, single-center, single-arm, open-label	Interventional	Apatinib, 500mg qd p.o.; Camrelizumab, 200mg every 3 weeks i.v.; oral S-1	Median PFS (months); Median OS (months)	PFS: 6.5; OS: NR
NCT03813784	III	Randomized, open-label, multi-center	Interventional	Apatinib, 250mg qd p.o.; Camrelizumab, 200mg every 3 weeks i.v.; Capecitabine; Oxaliplatin	Median PFS (months); Median OS (months); ORR (%)	/

PFS, progression-free survival; OS, overall survival; ORR, overall response rate; DCR, disease control rate; pRR, Pathologic response rate; NR, data not reached; NA, data not available.

## Clinical Efficacy in Liver Cancer and Cholangiocarcinoma

### Monotherapy or Combination Transarterial Therapy of Apatinib

The AHELP study, as a randomized, multicenter, double-blind, placebo-controlled, phase III trial (NCT02329860), was completed to evaluate the efficacy and safety of apatinib in pretreated patients with advanced hepatocellular carcinoma who had been refractory or intolerant to previous first-line systemic chemotherapy (oxaliplatin-based chemotherapy), targeted therapy (such as sorafenib), or both. A total of 400 eligible patients were randomly assigned to 2:1 groups receiving apatinib 750 mg (n = 267) or placebo (n = 133) orally once daily for a 28-day treatment cycle. Compared with the placebo group, the median OS in the apatinib group was significantly improved as the primary endpoint (8.70 months, 95% CI, 7.5 to 9.8 months versus 6.8 months, 95% CI, 5.7 to 9.1; hazard ratio 0.785, 95% CI, 0.617 to 0.998) and the median PFS was notably increased in comparison to placebo (4.5 months, 95% CI, 3.9 to 4.7 months versus 1.9 months, 95% CI, 1.9 to 2.0; hazard ratio 0.471, 95% CI, 0.369 to 0.601). 257 patients in the apatinib group and 130 patients in the placebo group received the safety assessment after the study treatment and thus the most common grade 3~4 adverse events were hypertension, hand-foot syndrome, and decreased platelet count. 37 patients (24 in the apatinib group and 13 in the placebo group) died due to adverse events, but the researchers believed that these deaths were not related to therapy. It was worth mentioning that the results of the AHELP trial led NMPA to approve apatinib as a second-line treatment for patients with advanced hepatocellular carcinoma on Dec 31, 2020 (51). Previous studies have shown that antiangiogenic drugs can promote the infiltration of immunosuppressive cells and enhance the expression of immune checkpoint molecules. Anti-VEGF therapy in combination with immunotherapy is a promising strategy to improve the treatment effect of patients with advanced primary liver cancer (52).

In China, most patients at an advanced stage of the disease cannot tolerate a radical surgery, and treatment of transcatheter arterial chemoembolization (TACE) can effectively prolong the survival time and increase the 2-year survival rate as a kind of the preferred non-surgical treatment. However, TACE usually cannot destroy all tumors, and the local hypoxia environment caused by embolization also stimulates tumor neovascularization (53). TACE combined with apatinib was proved to be an effective and safe way for patients with advanced hepatocellular carcinoma from the results of representative studies. In a total of 125 patients with TACE refractory intermediate or advanced-stage study, the median PFS of the TACE-apatinib group and TACE alone group were 7.0 and 2.0 months, while the median OS was 17.0 and 10.7 months, respectively (54). The median time of tumor progression (TTP) and OS of the TACE-apatinib group in another study were also significantly higher than those of the TACE monotherapy group after PSM analysis (TTP: 7.0 months versus 3.0 months; OS: 13.0 Months versus 8.0 months) (55).

Cholangiocarcinoma is one of the highly malignant tumors with a poor prognosis and approximately 60~70% of patients who are diagnosed with advanced stages are not suitable for surgical resection. Although gemcitabine combined with cisplatin or 5-fluorouracil is the first-line treatment to improve survival, the median OS is still shorter than 1 year. The fact that no standard second-line and higher therapy plan suggests that new treatment methods are urgently needed to establish to improve the survival of patients (56). There was a prospective open-label phase II trial (NCT03521219) to assess the clinical efficacy and safety of apatinib in 32 patients with advanced cholangiocarcinoma after the failure of gemcitabine-based chemotherapy. Patients were administered a second-line monotherapy of orally 500 mg apatinib per day for 28 days as a cycle until the disease progressed or unacceptable toxicity appeared. The primary endpoint of the ORR was 20.8% (95% CI, 9.24 to 40.47%) and the DCR was 62.5% (95% CI, 42.71 to 78.84%), meanwhile, the median PFS was 95 days (95% CI, 79.70 to 154.34 days) and the median OS was 250 days (95% CI, 112.86 to 387.14 days). Regarding safety, the most common clinical adverse events, mainly grade 1 or 2, included bone marrow suppression (69.2%), hypertension (57.7%), and proteinuria (46.2%) with no death caused by toxicity (57).

### Combination Immunotherapy of Apatinib

The RESCUE study (NCT03463876), conducted by the same author mentioned above, enrolled 70 first-line patients and 120 second-line patients with advanced hepatocellular carcinoma receiving 200 mg intravenous camrelizumab every two weeks plus 250 mg oral apatinib every day in 4-week cycles based on the results of the phase I study (46). As a non-randomized, open-label, multicenter, phase II study, the ORR was 34.3% (95% CI, 23.3 to 46.6%) in the first-line group and 22.5% (95% CI, 15.4 to 31.0%) in the second-line group. The median PFS in the two cohorts was 5.7 months (95% CI, 5.4 to 7.4 months) and 5.5 months (95% CI, 3.7 to 5.6 months), respectively. The 12-month OS were 74.7% (95% CI, 62.5 to 83.5%) and 68.2% (95% CI, 59.0 to 75.7%), respectively. The grade 3 treatment-related adverse events were reported in 147 (77.4%) of patients with hypertension (34.2%). Two patients (1.1%) had treatment-related deaths occurred. This manageable phase II study demonstrated that camrelizumab combined with apatinib represented durable responses, long survival, high ORR, and controllable safety in advanced HCC (58). In a cohort, multicenter phase Ib/II trial (NCT03092895), 28 patients were enrolled to receive 3 mg/kg camrelizumab (once every 2 weeks) plus different doses of apatinib (125, 250, 375, or 500 mg; once daily) during a 3 + 3 dose-escalation phase and subsequent expansion phase with the tolerability and safety as the primary endpoint. The 375 mg cohort was expanded due to reported two dose-limiting toxicities, grade 3 diarrhea in the 500 mg cohort. 8 patients of the 19 patients in the 375 mg cohort reduced apatinib dose to 250 mg within 2 months and 26 patients of 28 patients had treatment-related grade 3~4 adverse events. The secondary endpoints included the median PFS of 3.7 months (95% CI, 2.0 to 5.8 months) and the median OS of 13.2 months (95% CI, 8.9 to NR months). In conclusion, a 250 mg dose of apatinib was recommended as a combination regimen for further studies with controllable toxicity and promising antitumor activity (59).

Similarly, considering the potential combinational effects of apatinib plus camrelizumab, it can be an effective treatment for patients with cholangiocarcinoma. A prospective, non-randomized, open-label phase II trial (NCT04642664) was conducted on a total of 22 patients who had received previously treatments with receiving apatinib 250 mg orally per day, camrelizumab 200 mg intravenously every three weeks until intolerable toxicity or disease progression happened. The results of the median OS of 13.1 months (95% CI, 8.1 to 18.2 months), the median PFS of 4.4 months (95% CI, 2.4 to 6.3 months), the ORR of 19.0%, and the DCR of 71.4% demonstrated that the regimen had favorable therapeutic effects. Moreover, treatment-related adverse events occurred in all patients, with 14 of 22 patients (63.6%) experiencing grade 3~4, and no death was observed (60). This is the first report on the efficacy and safety of camrelizumab in combination with apatinib in patients with cholangiocarcinoma. Most studies on immunotherapy for advanced biliary tract cancer are in the exploratory stage, and these findings demonstrated promising therapeutic efficacy and relatively manageable toxicity, which needs further trials (61, 62).

Selected clinical trials summarized in **Table 2** highlighted that PD-1/VEGFR-targeted therapy can maintain efficacy after sorafenib exposure, suggesting no cross-reaction in the two therapeutic approaches for the first time (26). Therefore, an international multicenter randomized phase III study of camrelizumab combined with apatinib (NCT03764293) is conducted in progress in the first-line treatment of advanced primary liver cancer.

**Table 2:** Selected clinical trials with apatinib in liver cancer and cholangiocarcinoma.

Clinical trial identifier	Phase	Design	Type	Treatment	Primary endpoint	Results
NCT02329860	III	Randomized, multicenter, double-blind, placebo-controlled	Interventional	Apatinib, 750 mg qd p.o. Placebo bid p.o.	Median PFS (months); Median OS (months)	PFS: 4.5; OS: 8.7
NCT03521219	II	Prospective, open-label	Interventional	Apatinib, 500 mg qd p.o.	Median PFS (months); Median OS (months); ORR (%); DCR (%)	PFS: 3.2; OS: 8.3; ORR: 20.8; DCR: 62.5
NCT03463876	II	Single-arm, non-randomized, open-label, multicenter	Interventional	Apatinib, 250mg qd p.o.; Camrelizumab, 200mg every 2 weeks i.v.	Median PFS (months); 12-month OS (%); ORR (%)	PFS: 5.7/5.5; OS: 74.7/68.2; ORR: 34.3/22.5
NCT03092895	II	Nonrandomized, cohort, open-label, multicenter	Interventional	Apatinib, 375 mg qd p.o.; SHR-1210, 3 mg/kg every 2 weeks i.v.	Median PFS (months); Median OS (months)	PFS: 3.7; OS: 13.2
NCT04642664	II	Prospective, non-randomized, open-label	Interventional	Apatinib, 375mg qd p.o.; Camrelizumab, 200mg every 3 weeks i.v.	Median PFS (months); Median OS (months); ORR (%); DCR (%)	PFS: 4.4; OS: 13.1; ORR: 19.0; DCR: 71.4
NCT03764293	III	Randomized, open-label, international, multicenter	Interventional	Apatinib, 250mg qd p.o.; Camrelizumab, 200mg every 2 weeks i.v.	Median PFS (months); 12-month OS (%)	/

PFS, progression-free survival; OS, overall survival; ORR, overall response rate; DCR, disease control rate; NA, data not available.

## Clinical Efficacy in Colorectal Cancer

### Monotherapy or Combination Therapy of Apatinib

The efficacy and safety of apatinib in chemotherapy-refractory patients with metastatic colorectal cancer were eligible for assessments in a single-arm, multicenter, phase II clinical trial (NCT03397199). The median PFS and OS of 29 patients who received oral apatinib (250 mg daily) combined with S1 (40-60 mg/d in days 1-14, for 3 weeks) were 7.9 months (95% CI, 4.9 to 10.9 months) and 12.9 months (95% CI, 9.6 to 16.2 months), respectively. Exploratory analysis showed that patients who took S-1 for ≥ 70 days achieved an ORR of 13.79% and a DCR of 89.66%. Ten grade 3 adverse events were reported and the incidence of each event was less than 5%. No grade 4 side effects were observed (63). The results of this apatinib combination clinical trial are more convincing than another multicenter, single-arm, prospective study of apatinib alone treatment without NCT number. Overall, 48 patients were given 500 mg apatinib once daily who had advanced progression after second-line or higher-line standard chemotherapy with a median PFS of 4.8 months (95% CI, 3.7 to 5.9 months) and the median OS of 9.1 months (95% CI, 5.2 to 13.1 months) (64). The results did not differ between subgroups and were consistent with the recently published trials of apatinib by a real-world retrospective study (PFS: 3.8 months, not reaching OS) and a pilot Study (PFS: 4.8 months, OS: 10.1 months) (65, 66). The common grade 3~4 adverse events were hypertension (6 of the 48 patients), hand-foot syndrome (5 of those), thrombocytopenia (5 of those), and proteinuria (4 of those), which were the main reasons for dose modification of apatinib (64). It was more likely to benefit from apatinib for patients with metastatic colorectal cancer who previously received antiangiogenesis therapy and had baseline elevated neutrophil to lymphocyte ratio (67). In the latest phase-II, single-arm, prospective study (NCT03210064), 16 patients who had failed at least 2 previous standard treatment regimens with pathologically colorectal cancer received apatinib 250mg for 28 consecutive days and 5-FU, resulting in the ORR was 25.0% (95% CI, 7.3 to 52.4%), and the DCR was 68.8% (95% CI, 41.3 to 89.0%). The median PFS was 4.83 months (95% CI, 2.2 to 8.9 months), and the median OS was 9.10 months (95% CI, 5.6 to 15.2 months). Grade 3 adverse events occurred in 7 patients, including hand-foot syndrome (18.75%), hypertension (12.50%), and proteinuria (12.50%) (68). The excellent anti-tumor effect of the combination regimen may be related to the multiple antitumor mechanisms of apatinib and the cytotoxicity of 5-FU (69).

### Combination Immunotherapy of Apatinib

Regarding combination therapy of the REGONIVO study (NCT03912857), a prospective, single-arm, open-label, phase II trial demonstrated that apatinib may improve the efficacy of camrelizumab in the treatment of microsatellite stable (MSS) metastatic colorectal cancer. 10 patients received 375 mg of apatinib orally once daily, and the dose of apatinib could be reduced to 250 mg for those who could not tolerate the toxicity. Camrelizumab is administered intravenously every two weeks at a dose of 200 mg. However, in this study, all patients (100%) had treatment-related adverse events with an ORR of 0%, a DCR of 22.2%, a median PFS of 1.83 months (95% CI, 1.80 to 1.86 months), and a median OS of 7.80 months (95% CI, 0 to 17.07 months). Apatinib combined with immunotherapy failed to improve the therapeutic effect while producing serious adverse effects. Reducing the dose of apatinib or combining anti-PD-1 antibodies with other well-tolerated antiangiogenic drugs may help to design new and better treatment strategies. The leading reason for failure was the intolerable toxicity to patients including hypertension and proteinuria (70% each) (70).

The selected clinical trials are found in **Table 3** that apatinib combined with chemotherapy achieved clinical effects in the treatment of advanced colorectal cancer to some extent, while unfortunately, the combination of immunotherapy has not seen the dawn of victory. This result is related to the fact that the sample size is small, hence more follow-up case reports are needed to support the evidence.

**Table 3:** Selected clinical trials with apatinib in colorectal cancer and esophageal squamous cell carcinoma.

Clinical trial identifier	Phase	Design	Type	Treatment	Primary endpoint	Results
NCT03397199	II	Single-arm, multicenter	Interventional	Apatinib, 250 mg qd p.o.; S-1	Median PFS (months); Median OS (months)	PFS: 7.9; OS: 12.9
NCT03210064	II	Randomized, single-arm, multicenter	Interventional	Apatinib, 250 mg qd p.o.; 5-fluorouracil derivatives	Median PFS (months); Median OS (months); ORR (%); DCR (%)	PFS: 4.83; OS: 9.10; ORR: 25.0; DCR: 68.8
NCT03912857	II	Prospective, single-arm, open-label	Interventional	Apatinib, 250 mg qd p.o.; SHR-1210, 200mg every 2 weeks i.v.	Median PFS (months); Median OS (months); ORR (%); DCR (%)	PFS: 1.83; OS: 7.80; ORR: 0; DCR: 22.2
NCT03736863	II	Single-arm, open-label	Interventional	Apatinib, 250 mg qd p.o.; SHR-1210, 200mg every 2 weeks i.v.	Median PFS (months); Median OS (months); ORR (%); DCR (%)	PFS: 6.8; OS: 15.8; ORR: 34.6; DCR: 78.8
NCT03603756	II	Prospective, single-group	Interventional	Apatinib, 250 mg qd p.o.; SHR-1210, 200mg every 2 weeks i.v.; Liposomal paclitaxel; Nedaplatin	Median PFS (months); Median OS (months); ORR (%); DCR (%)	PFS: 6.85; OS: 19.43; ORR: 80; DCR: 96.7

PFS, progression-free survival; OS, overall survival; ORR, overall response rate; DCR, disease control rate.

## Clinical Efficacy in Esophageal Squamous Cell Carcinoma

### Combination Immunotherapy of Apatinib

For patients with esophageal squamous cell carcinoma who had failed current first-line chemotherapy, apatinib combined with S-1, paclitaxel, or paclitaxel/capecitabine had demonstrated promising outcomes (71–73). The effective and well-tolerated combination therapy had the potential to be a potent therapeutic regime. However, the lack of registered NCT number and relatively small sample size required more investigative and complete clinical trials to validate the findings.

Recent a multicenter study (NCT03736863) of single-arm, open-label phase II immunotherapy combination in patients with unresectable locally recurrent, locally advanced, or metastatic esophageal squamous cell carcinoma who were intolerant to or had progressed after the first-line chemotherapy. A total of 52 patients received camrelizumab 200 mg intravenously every 2 weeks plus oral apatinib 250 mg for 28 days. In the full analysis set, the ORR was 34.6% (95% CI, 22.0 to 49.1%) and the DCR was 78.8% (95% CI, 65.3 to 88.9%). The median PFS was 6.8 months (95% CI, 3.8 to 10.4 months) and the median OS was 15.8 months (95% CI, 8.4 to 16.2 months). 23 (44%) of 52 patients had treatment-related grade 3-4 adverse events including increased gamma-glutamyltransferase (19%), increased aspartate aminotransferase (19%), and increased alanine aminotransferase (10%) (74). As a potential second-line treatment provided an option for patients with advanced esophageal squamous cell carcinoma and the safety was controllable. A phase III clinical study is needed to verify the promising benefit of this combination. In a three-drug combination single-arm prospective phase II trial (NCT03603756) of camrelizumab combined with apatinib and chemotherapy, patients with unresectable locally advanced or recurrent/metastatic esophageal squamous cell carcinoma received 200 mg camrelizumab, 150 mg/m^2^ liposomal paclitaxel and 50 mg/m^2^ nedaplatin on day 1, and 250 mg apatinib on days 1-14. The results of ORR in 30 patients was 80.0% (95% CI, 61.4-92.3%) and the DCR reached 96.7% (95% CI, 82.8-99.9%). The median PFS was 6.85 months (95% CI, 4.46-14.20 months) and the median OS was 19.43 months (95% CI, 9.93 months - not reached). The most common grade 3-4 treatment-related adverse events were leukopenia (83.3%) and neutropenia (60.0%) (75). Camrelizumab combined with apatinib, liposomal paclitaxel, and nedaplatin as first-line therapy showed feasible antitumor activity and manageable safety in this patient population. In the near future, it will be necessary to evaluate this new combined approach in a randomized phase III trial. All selected clinical trials are found in **Table 3**.

## Molecular Mechanisms

In clinical trials, apatinib showed comparable survival results, meanwhile, the observed promising efficacy of apatinib may be due to the following reasons. First, apatinib has highly selective competition in the tyrosine kinase activity of VEGFR-2, the most significant receptor in tumor pathological conditions, then blocking downstream VEGF-mediated endothelial cell proliferation and inhibiting tumor angiogenesis (76). Anti-proliferative as a kind of important feature in apatinib has been addressed by researchers. For example, Zhijian Jin et al. have investigated that in anaplastic thyroid carcinoma cells line treatment of apatinib reduced the expression of angiopoietin and inhibited the angiogenesis *in vivo* and vitro. Apatinib inhibited cell growth by inducing apoptosis and blocking cell cycle progression G0/G1 phase in a dose-dependent manner by inducing suppression of AKT/GSK-3β/ANG signaling pathway (77). He K et al. showed the biological function of apatinib in pancreatic cancer cells line including inhibiting the expression of hypoxia-inducible factor-1α (HIF-1α) and markers of the PI3K/AKT/mTOR signaling pathway was able to reduce the proliferation (78). Second, apoptosis and autophagy are the two main mechanisms leading to programmed cell death. Up-regulation of autophagy and apoptosis may lead to the death of tumor cells. Haoran Feng et al. examined that apatinib down-regulated p-AKT and p-mTOR signals through the AKT/mTOR pathway and induced autophagy and apoptosis in human anaplastic thyroid carcinoma cells line. In addition, inhibition of apatinib-induced autophagy increased apoptosis, and the combination of apatinib and the autophagy inhibitor chloroquine produced a promising tumor suppression effect *in vivo* and *in vitro* (31). Meanwhile, Lu et al. firstly discovered that in colorectal cancer cell lines apatinib could induce autophagy *in vitro* but no further mechanisms had been found (79). Interestingly, a novel antitumor effect of pyroptosis was identified by Qiwu Zhao et al. in the combined application of apatite and melittin for patients with anaplastic thyroid carcinoma. Mechanistically, upregulated cleaved caspase-1 activated caspase-3 by generating GSDMD-N terminal, while cleaved caspase-3 enhanced caspase-1 activation which could induce pyroptosis by GSDME-N and vice versa. These processes constitute a unique system involving two-way positive feedback regulation (80). Our previous study indicated that after apatinib treatment ER stress could induce autophagy in colorectal cancer cell lines through the IRE1α pathway. Moreover, the autophagy activation induced by apatinib treatment had a protective effect on the colorectal cancer cells line. The combination of chloroquine and siRNA targeting Atg5 to block autophagy could significantly drive the apoptosis process. Therefore, the use of chloroquine combined with apatinib therapy tended to have a better significant tumor suppression (81). An interesting mechanism recently reported for gastric cancer xenograft models through microRNA and circular RNA-sequencing analysis was proved by Ling Ma et al. that circRACGAP1 acted as an endogenous sponge of miR-3657 to block its activity and then upregulated ATG7 expression to promote autophagy activation. Moreover, the knockdown of circRACGAP1 *in vitro* and *in vivo* by autophagy inhibition made gastric cancer cell lines sensitive to apatinib (82). Further research is needed to provide more comprehensive and detailed evidence.

**Figure 1:**
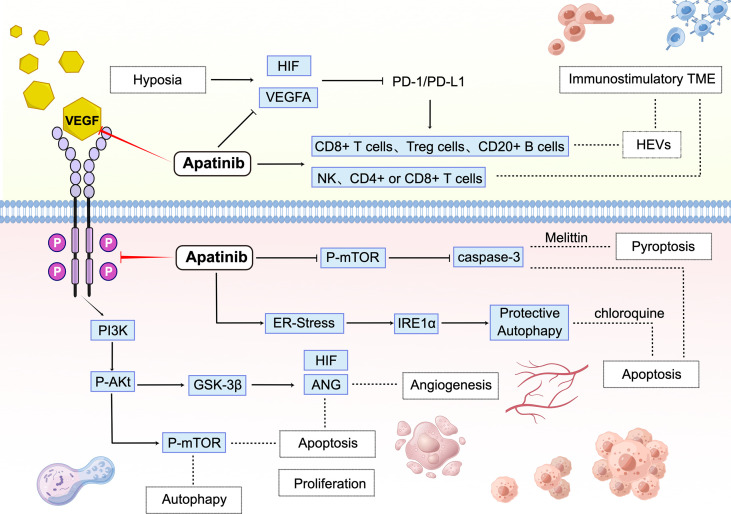
Molecular mechanisms involved in the antitumor effect of apatinib monotherapy and combination immunotherapy.

As previously mentioned, shreds of evidence have been provided that angiogenesis-induced immunosuppression can be used to improve immunotherapy from numerous preclinical studies (83). Therefore, the treatment of adding antiangiogenic drugs to immunotherapy is considered to be attractive. First, the effective antitumor response of PD-1 blockade is mainly dependent on the ability of specific T cells to infiltrate tumor areas. Abnormal tumor vasculature often produces hypoxia and acidic TME to interfere with drug penetration in tumor (84, 85). Hypoxia further promotes angiogenesis, epithelial-mesenchymal transition, and tumor metastasis through vascular endothelial growth factor A (VEGFA) or HIF-1, exacerbating hypoxia and immunosuppression, and patients are less likely to benefit from this therapy (86, 87). In immunosuppressive TME, apatinib may show a promising immunomodulatory activity. Second, sufficient clinical data support that inhibition of the VEGFA signaling pathway may improve T-cell-mediated antitumor immunity (88, 89). However, Yinli Yang et al. proposed that in a syngeneic mouse model of liver cancer, the natural killer cells, but not CD4+ or CD8+ T cells, mediate the therapeutic effect of apatinib. In addition, apatinib not only effectively inhibited tumor growth and angiogenesis in liver cancer but also induced NK cell activation, increased levels of interferon-γ, and decreased levels of tumor necrosis factor-α and interleukin-6, suggesting the potential benefit of combination therapy with PD-1 blockade and apatinib in liver cancer (90). Third, high endothelial venules (HEVs) can arise spontaneously as anatomically postcapillary venules found in cancer (91) and are often associated with good clinical outcomes (92–96). Preliminary exploration by Yu Zhang et al. demonstrated an association between clinical outcomes and HEVs in several solid tumors. The role of HEVs was associated with lymphocyte infiltration, immune targeting, and response to targeted therapy. The combination of apatinib and PD-L1 blockade increased the ratio of CD8^+^ cytotoxic T cells to Foxp3+ Treg cells, accumulation of CD20^+^ B cells, and Th1/Th2 cytokine ratio to promote antitumor immunity. Combination therapy induced HEVs formation by activating LTβR signaling, thus promoting the infiltration of CD8^+^ cytotoxic T cells and CD20^+^ B cells in the tumor. The results synergistically delayed the tumor growth and improved survival in gastric cancer mice (97). Anti-angiogenesis can prevent metastasis, improve immunotherapy, enhance drug penetration, and reshape the TME (23, 98, 99). In this way, apatinib combined with immunotherapy has a molecular theoretical basis in **Figure 1**.

Apatinib inhibits the tumor cells’ proliferation, angiogenesis, and apoptosis through VEGFR-2/PI3K/AKT/GSK3β. Moreover, apatinib stimulates apoptosis, autophagy, and pyroptosis *via* PI3K/AKT/mTOR and ER-stress/IRE1α. In TME, a hypoxic environment leads to tumor resistance to PD-1/PD-L1, while apatinib can inhibit HIF/VEGFA to increase the sensitivity of immune checkpoint blockade agents and activate NK、CD4^+^ or CD8^+^ T cells. CD8^+^ T cells, Treg cells, and CD20^+^ B cells can be activated by combination therapy to increase tumor-associated HEVs, thus reshaping it an immunostimulatory TME. PI3Ks, Phosphoinositide 3-kinases; GSK-3β, Glycogen synthase kinase 3 beta; ANG, Angiogenin; HIF, Hypoxia-inducible factor; mTOR, Mammalian target of rapamycin; IRE1α, Inositol-requiring enzyme-1α.

**Figure 2:**
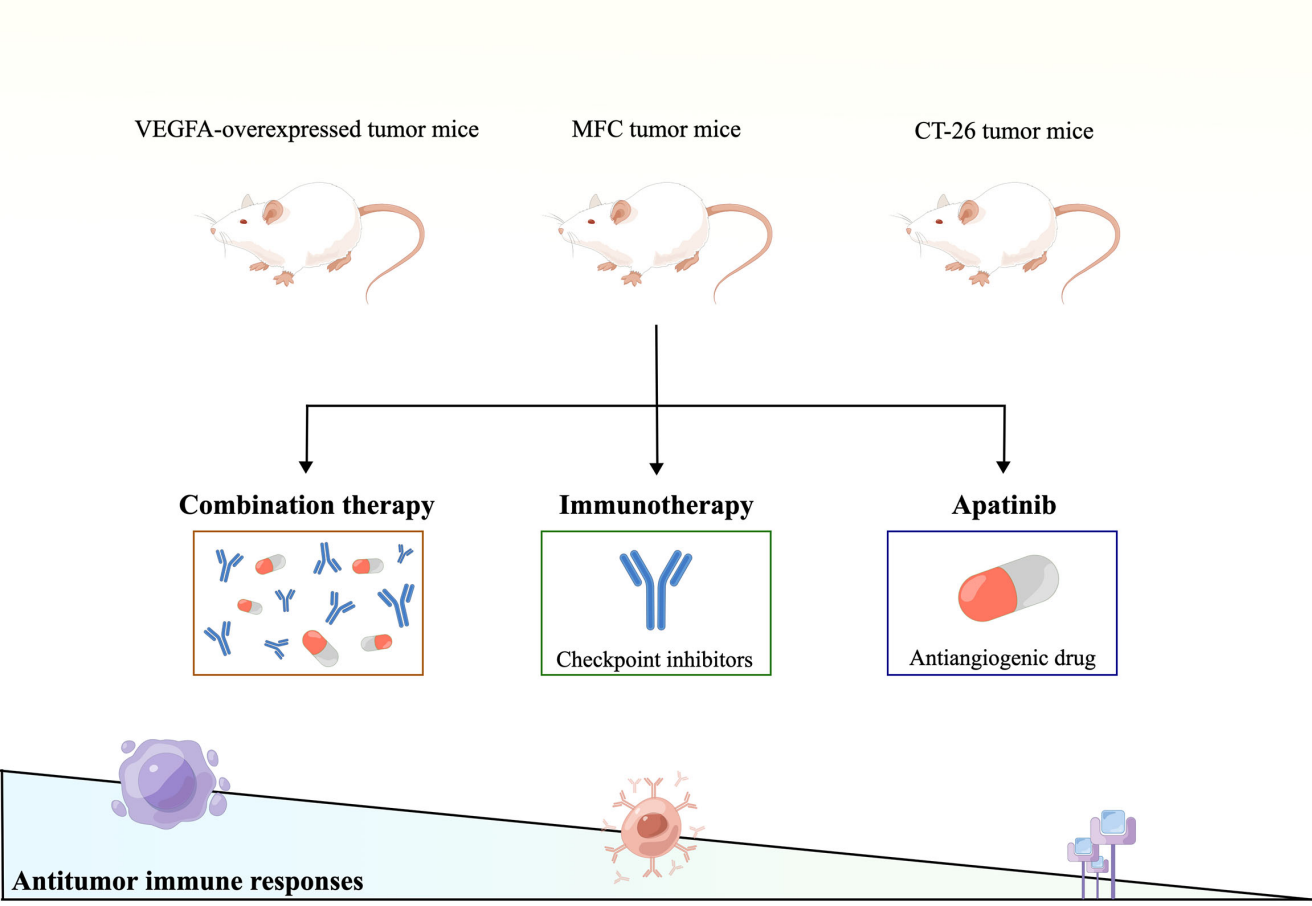
Therapeutic strategies of apatinib combined with immune checkpoint inhibitors to match the expanding gradient of systemic therapeutic responsiveness.

Several excellent preclinical studies have shown that inhibition of angiogenesis, alone or in combination with different immunotherapies, improves antitumor immunity. For example, the VEGFA-overexpressed mice breast and colon subcutaneous models characterized by hypoxia, hyper-angiogenesis, and immunosuppressive TME were established, which displayed innate resistance to immunosuppressive agents. In the VEGFA-overexpressed TME, apatinib attenuated excessive angiogenesis and hypoxia and converted the immunosuppressive TME to an immunostimulatory TME. In contrast, no beneficial phenomenon was observed in tumor-bearing mice without VEGFA overexpression. The combination of apatinib and immune checkpoint inhibitory caused T cell exhaustion, downregulated inhibitory receptors’ co-expression, and reduced suppressive immune cells’ accumulation, including regulatory T cells (Tregs) (100). These findings indicate that combination immunotherapy can be applied in a specific population, which might bring new inspiration to immunotherapy ineffective patients with colorectal cancer. In another immunocompetent mice model of subcutaneous MFC tumors study, combined treatment with apatinib and PD-L1 blockade synergistically delayed tumor growth and improved survival rates in MFC tumor-bearing immunocompetent mice. It is explained that combined apatinib and PD-L1 blockage treatment synergistically promotes HEV formation and enhances antitumor immune responses in gastric cancer (97). Further, in the co-culture system, apatinib-treated cancer cells upregulated PD-L1 expression and angiogenesis inhibition, and hindered T cell activation and IFN-γ secretion, which was reversed by an anti-PD-1 antibody. The anti-PD-1 antitumor efficacy was enhanced in a mouse model (CT-26 cells in Balb/c). The combination therapy had a more significant inhibitory effect on the growth of allograft tumors in mice than monotherapy (101). Taken together, these studies shown in **Figure 2** demonstrate that antiangiogenic drugs can improve intratumoral infiltration and activation of effector T cells, thus enhancing the efficacy of immunotherapy.

## Conclusion and Future Perspective

The efficacy and safety of apatinib in the treatment of advanced gastric cancer and GEJ carcinoma had been confirmed by NMPA in 2014. Meanwhile, several clinical trials based on the molecular mechanisms are in progress to explore the application in other solid tumors currently. In this review, we summarized the results of apatinib treatment in clinical trials and clarified the promising improvement in the treatment of DSMs. In the third-line or subsequent treatment of advanced gastric cancer, compared with placebo, oral apatinib can significantly prolong the median PFS and OS with manageable and tolerable safety from these clinical researches. The AHELP study concluded above demonstrated that apatinib achieved a comparable effect in patients with advanced hepatocellular carcinoma as a second-line treatment approved by NMPA on Dec 31, 2020. Exploratory studies also showed that apatinib had competitive efficacy in cholangiocarcinoma, colorectal cancer, and esophageal squamous cell carcinoma, thus the following large-scale phase III clinical trials were required for detailed verification. Growing evidence indicated that the combination of apatinib and some other invasive or non-invasive treatments could convert initially unresectable advanced tumors into resectable tumors. Up to now, immune checkpoint blockade has been an attractive option, especially for patients with advanced or metastatic tumors, while only a part of patients benefits from it. For patients with advanced gastric cancer, the combination of apatinib and camrelizumab can improve median PFS and median OS notably. The result is better than that of any single drug, especially the median OS, which extended from four months to more than ten months. As the most promising second-line targeted therapy for liver cancer, although there is no significant difference in PFS compared with apatinib monotherapy, immune checkpoint blockade agents greatly improve overall survival time and enhance the ORR and the DCR status to varying degrees. The failure of safety and efficacy of apatinib combined with immunotherapy in colorectal cancer limits the wide range of combination immunotherapy applications. The main reason for failure was the intolerable toxicity and the restricted sample size. The combination of immune checkpoint inhibitors and antiangiogenic drugs has surprising effects in esophageal squamous cell carcinoma, while it still needs to be further confirmed by large-scale, multicenter, randomized phase III clinical trials. The molecular mechanisms of combination therapy include reshaping immunosuppressive TME into immunostimulatory TME, improving NK and T-cell-mediated antitumor immunity, and inducing HEVs formation, thus synergistically improving survival and inhibiting tumor proliferation. It would be plausible to sustain that the angiogenesis process is an important driver of DSMs, meanwhile, it is reasonable to become the optimal target according to the mechanism. Some latest findings of the direct functional mechanisms of apatinib in inhibiting multiple TRKs and related pathways were discussed above. As a result, the anti-proliferation, apoptosis-inducing, and autophagy-inducing properties were explored in the corresponding signaling pathway. However, future research efforts to gain a deeper understanding of the mechanism of apatinib, a new oral VEGFR-2 inhibitor, are still important to improve the survival time of patients with DSMs and find better responders. To date, some standard second-line treatments for DSMs are still controversial. As a promising treatment regimen, combination usage with other drugs and the optimal dose in the treatment of other types of DSMs is required to be determined in further clinical studies and long-term pharmacovigilance. It is also significant to identify predictive biomarkers to select the best candidates based on big data in anti-angiogenesis therapies. In all, given the features of a convenient administration regimen with a good prognosis and limited treatment options, apatinib remains an optimal choice for patients with DSMs in the future.

## Author Contributions

LH, HH, and ZT were responsible for the primary review of literature and writing. FH, WS, and ZY analyzed and summarized the information. JX, CX, and ZR guided and supervised this study. All authors listed made a substantial, direct, and intellectual contribution to the work and approved it for publication.

## Funding

The study was supported by the Shanghai Science and Technology Commission, 18ZR1424300 (R.Z.); Shanghai hospital development center, SHDC2020CR1026B (R.Z); Shanghai Health Commission, 2019SY058 (R.Z.); National Natural Science Foundation of China, 82002475 (X.C.); Shanghai sailing program, 20YF1427700 (X.C.).

## Conflict of Interest

The authors declare that the research was conducted in the absence of any commercial or financial relationships that could be construed as a potential conflict of interest.

## Publisher’s Note

All claims expressed in this article are solely those of the authors and do not necessarily represent those of their affiliated organizations, or those of the publisher, the editors and the reviewers. Any product that may be evaluated in this article, or claim that may be made by its manufacturer, is not guaranteed or endorsed by the publisher.
